# Supplementary figures and images for: Sequence characteristics and phylogenetic analysis of H9N2 subtype avian influenza A viruses detected from poultry and the environment in China, 2018
Source: PeerJ. 2021 Dec 20;9:e12512. doi: 10.7717/peerj.12512 (PMC8697764; doi:10.7717/peerj.12512)

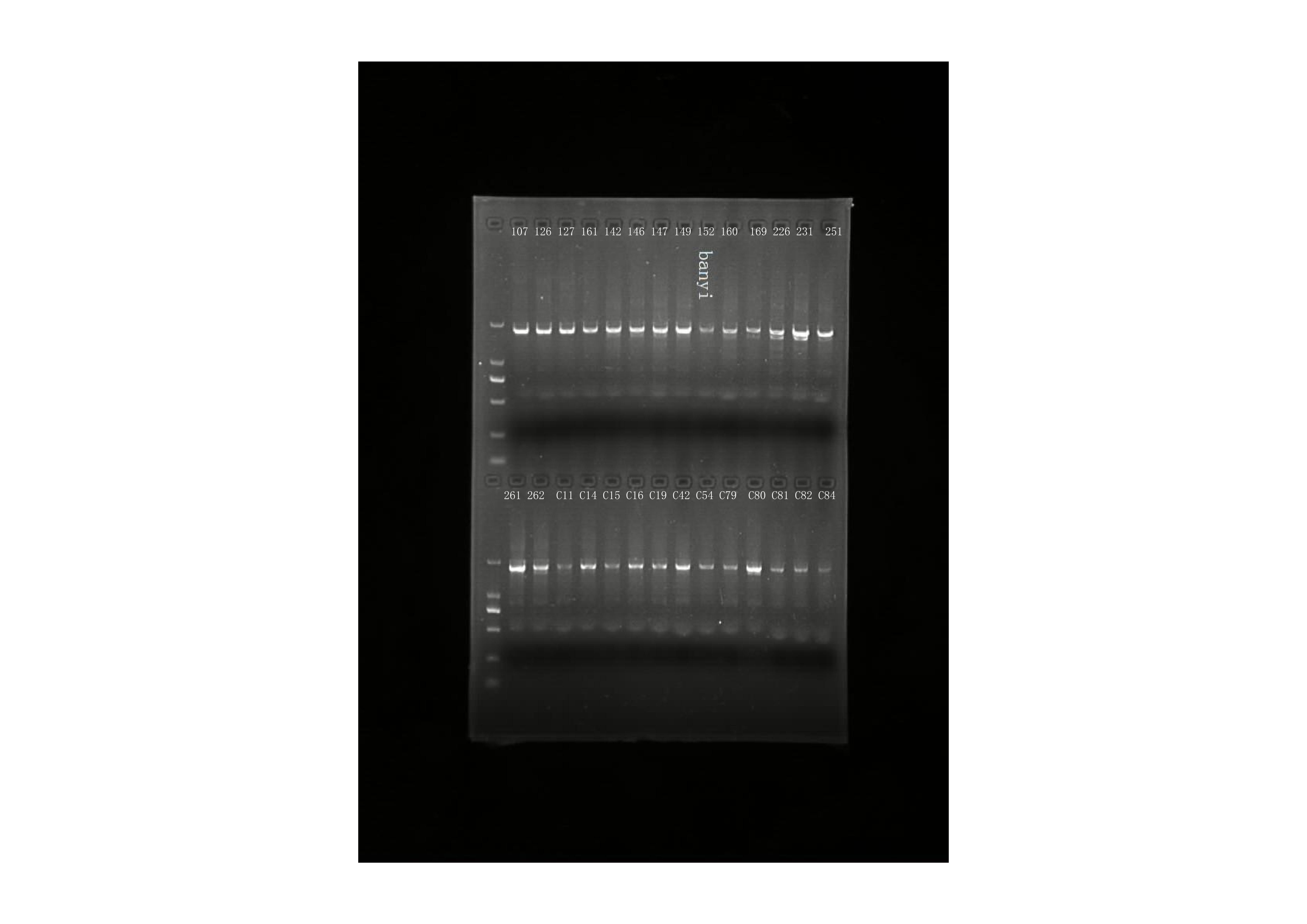

Supplement: Supplemental Information 1 — Raw data: result of RT-PCR amplificaiton of the ORF of HA gene. The name of sample was indicated in the picture. [file peerj-09-12512-s001.jpg]

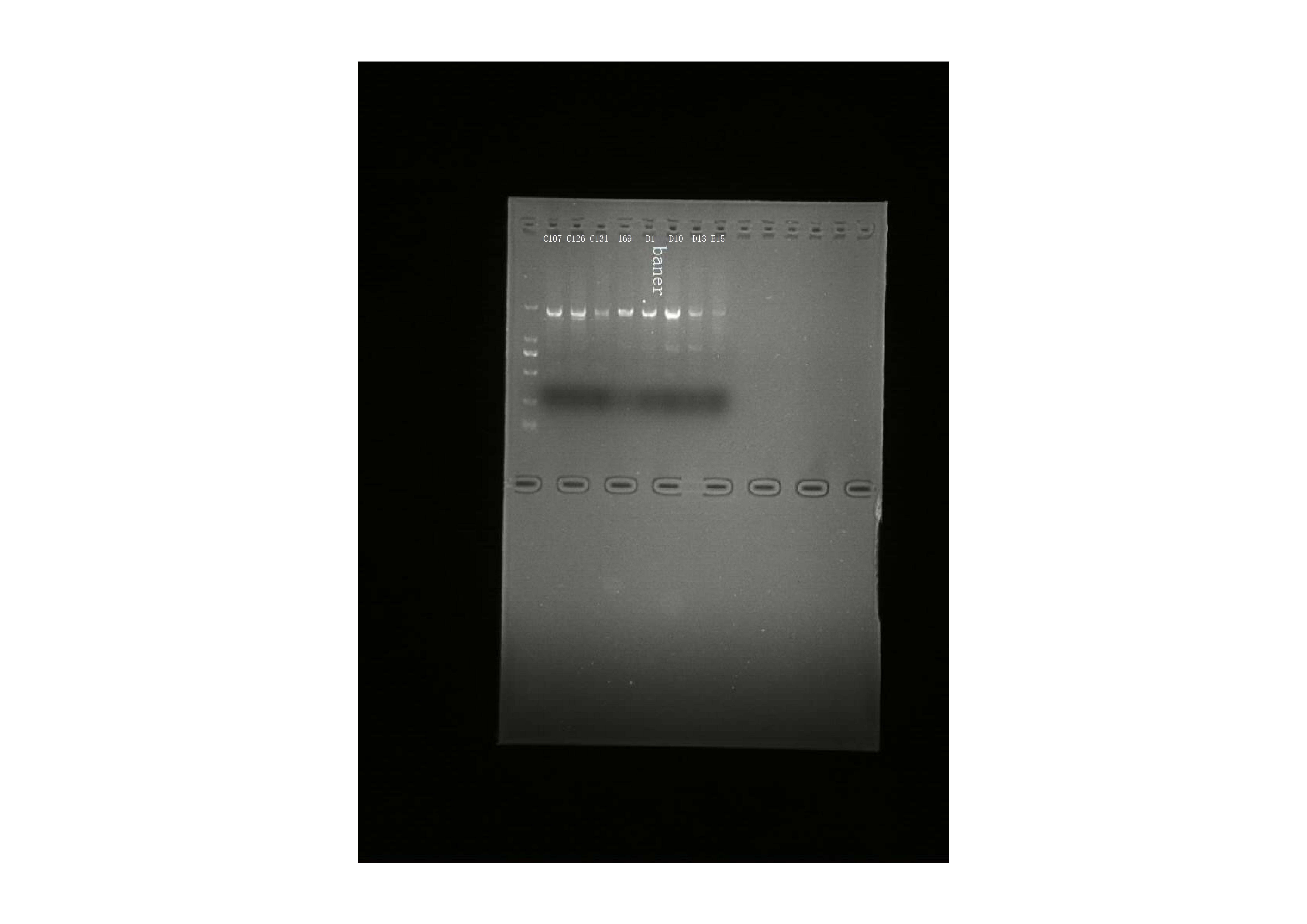

Supplement: Supplemental Information 2 — Raw data: result of RT-PCR amplificaiton of the ORF of HA gene. The name of sample was indicated in the picture. [file peerj-09-12512-s002.jpg]

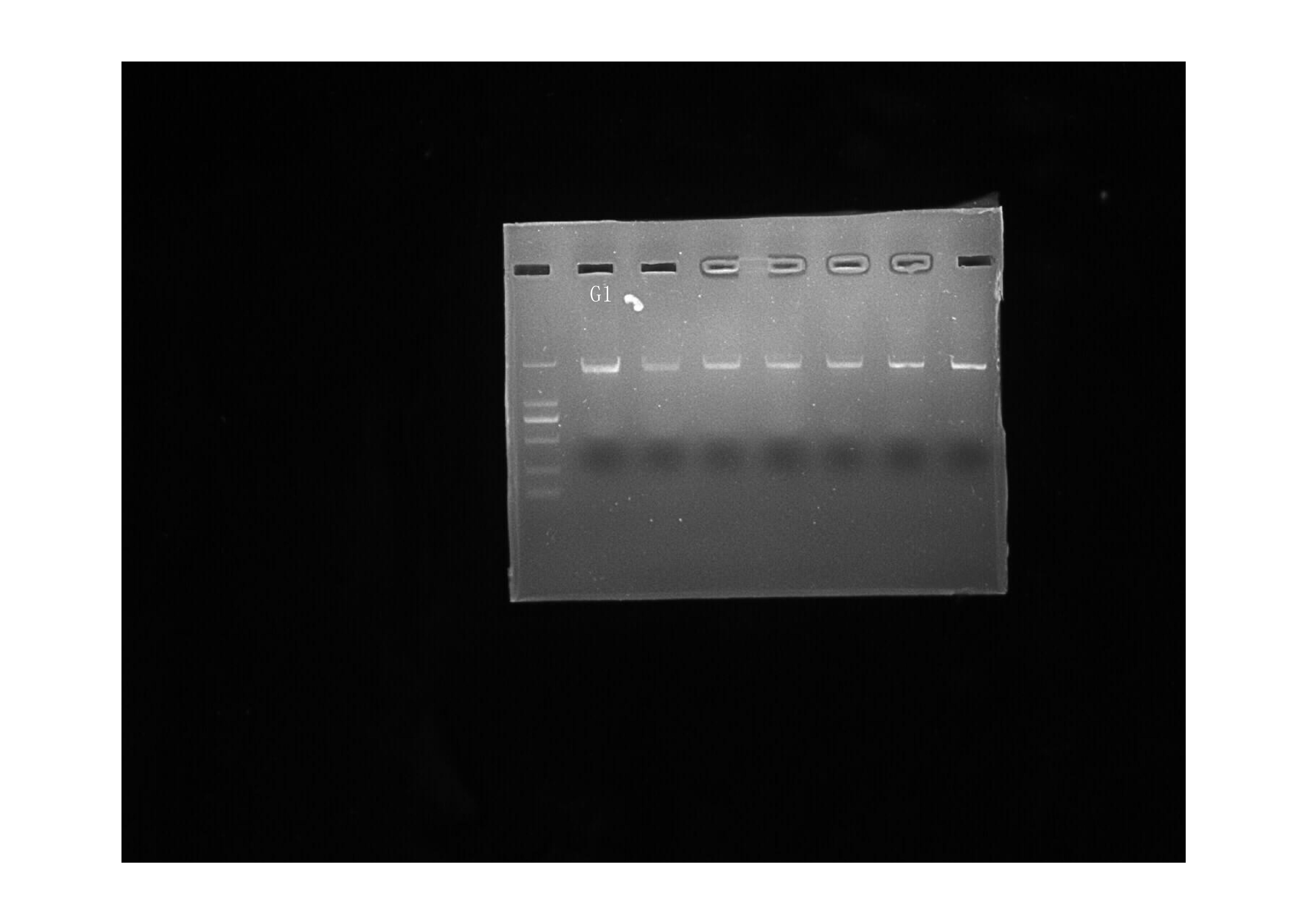

Supplement: Supplemental Information 3 — Raw data: result of RT-PCR amplificaiton of the ORF of HA gene. The name of sample was indicated in the picture. [file peerj-09-12512-s003.jpg]

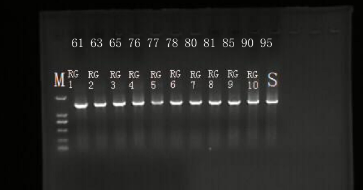

Supplement: Supplemental Information 4 — Raw data: result of RT-PCR amplificaiton of the ORF of HA gene. The name of sample was indicated in the picture. [file peerj-09-12512-s004.png]

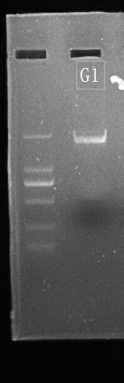

Supplement: Supplemental Information 5 — Raw data: result of RT-PCR amplificaiton of the ORF of HA gene. The name of sample was indicated in the picture. [file peerj-09-12512-s005.png]

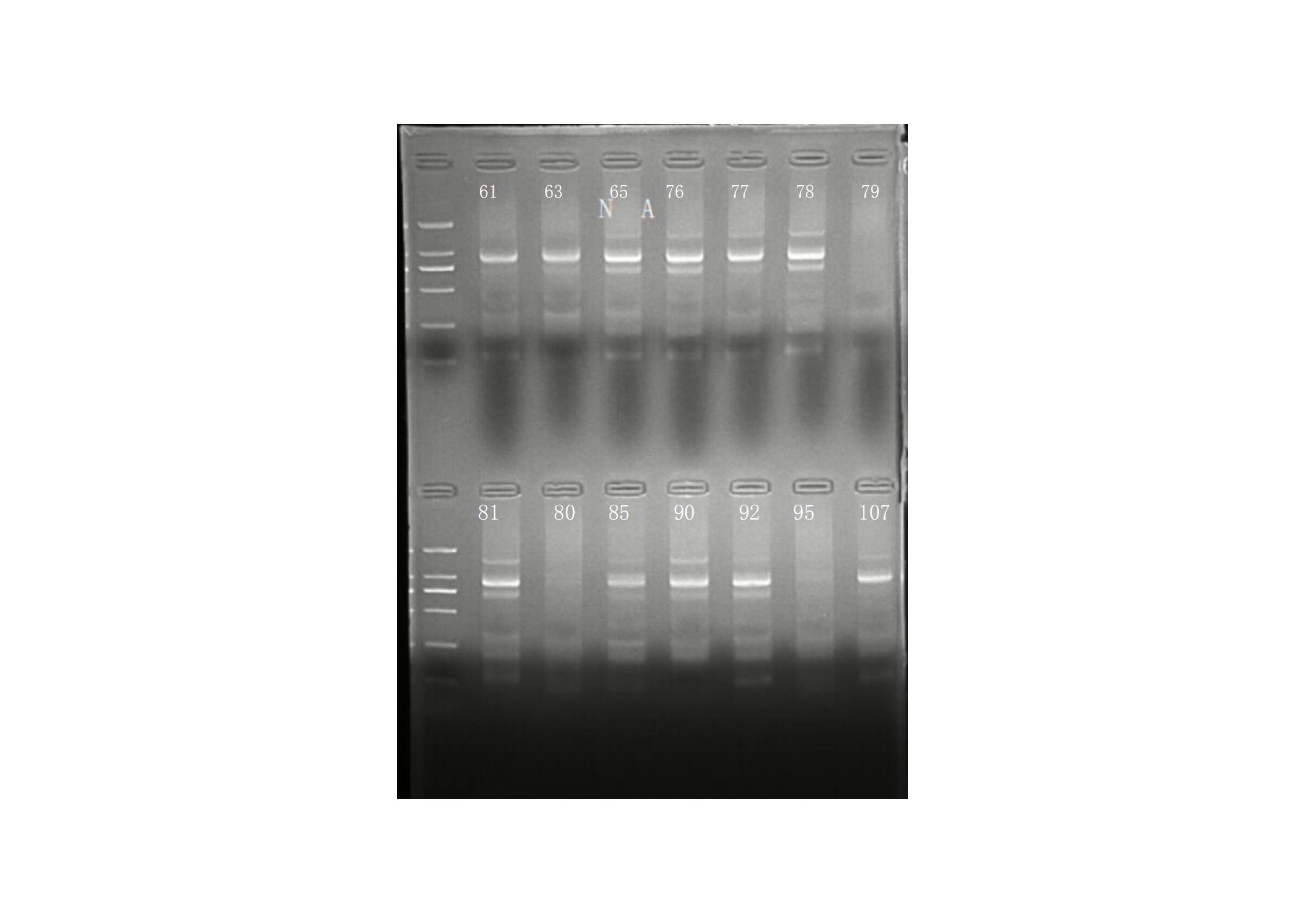

Supplement: Supplemental Information 6 — Raw data: result of RT-PCR amplificaiton of the ORF of NA gene. The name of sample was indicated in the picture. [file peerj-09-12512-s006.jpg]

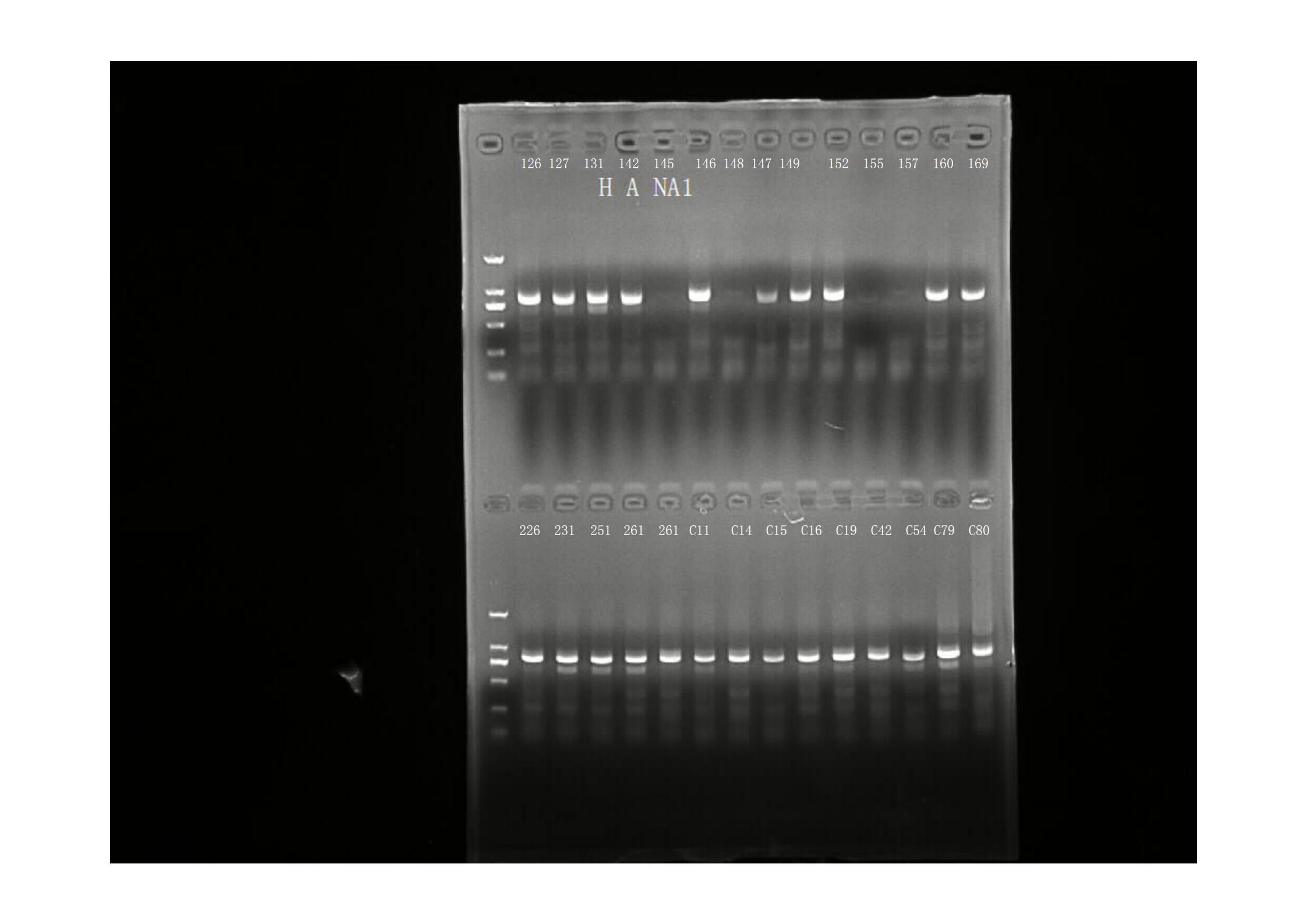

Supplement: Supplemental Information 7 — Raw data: result of RT-PCR amplificaiton of the ORF of NA gene. The name of sample was indicated in the picture. [file peerj-09-12512-s007.jpg]

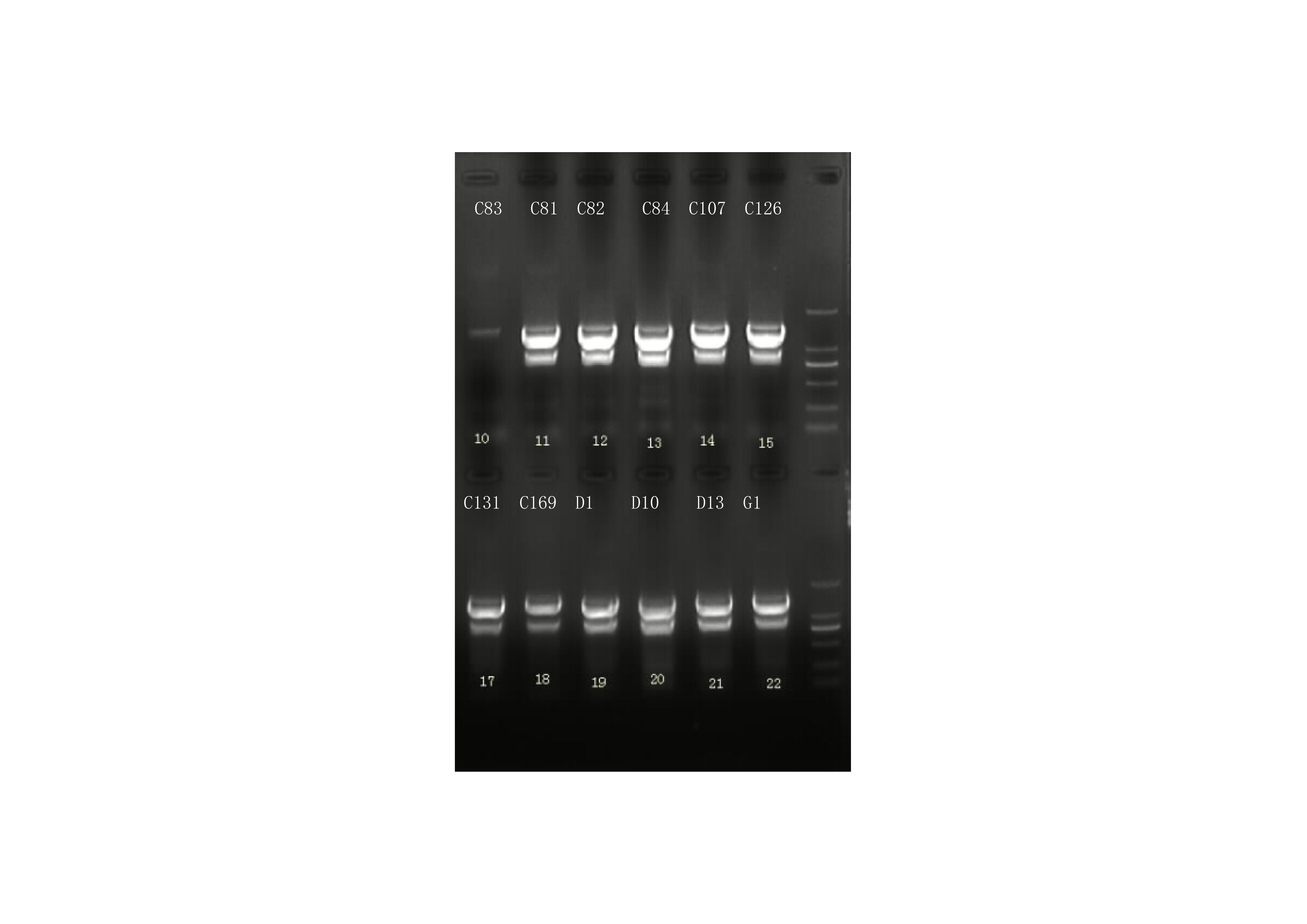

Supplement: Supplemental Information 8 — Raw data: result of RT-PCR amplificaiton of the ORF of NA gene. The name of sample was indicated in the picture. [file peerj-09-12512-s008.jpg]

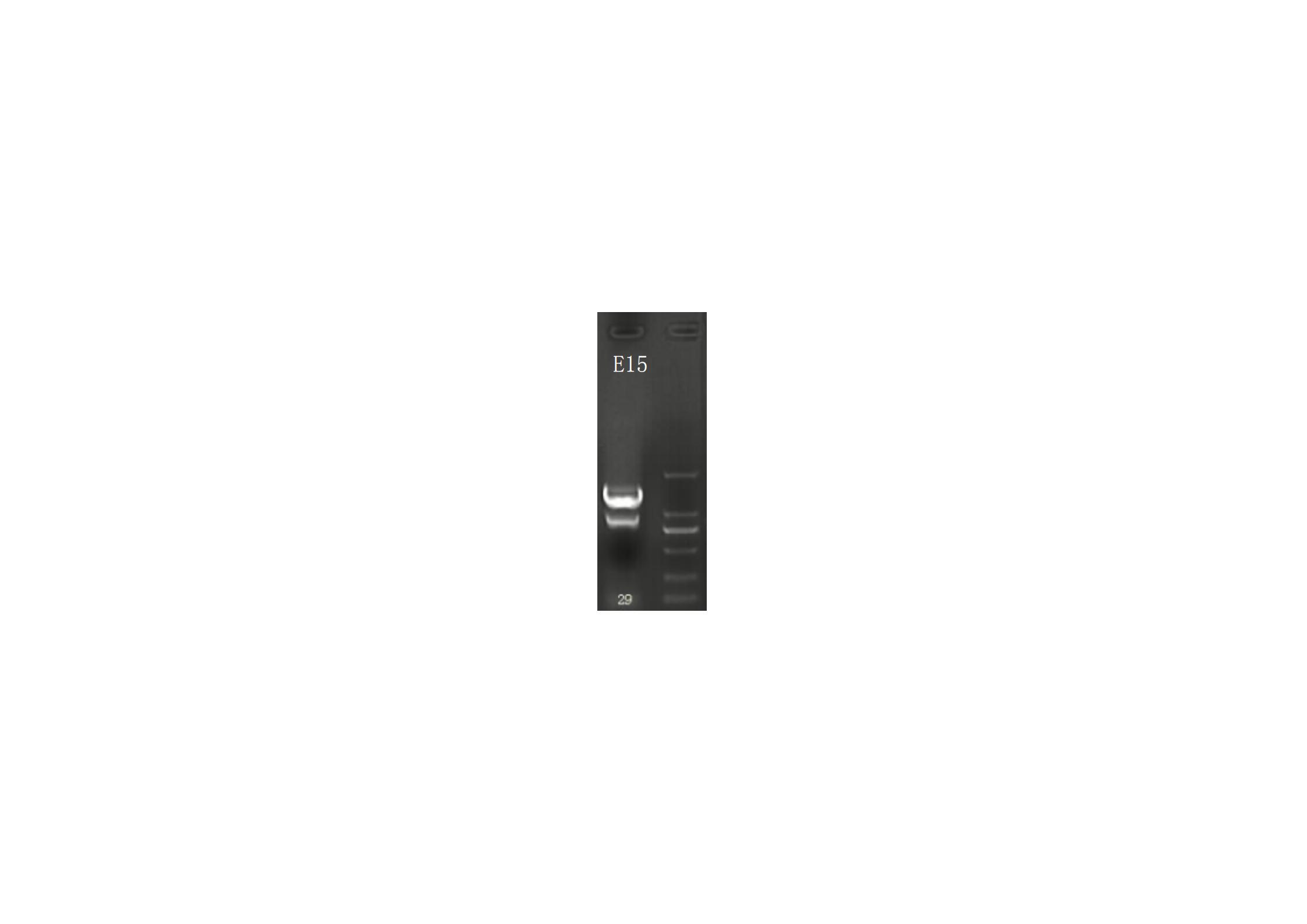

Supplement: Supplemental Information 9 — Raw data: result of RT-PCR amplificaiton of the ORF of NA gene. The name of sample was indicated in the picture. [file peerj-09-12512-s009.jpg]

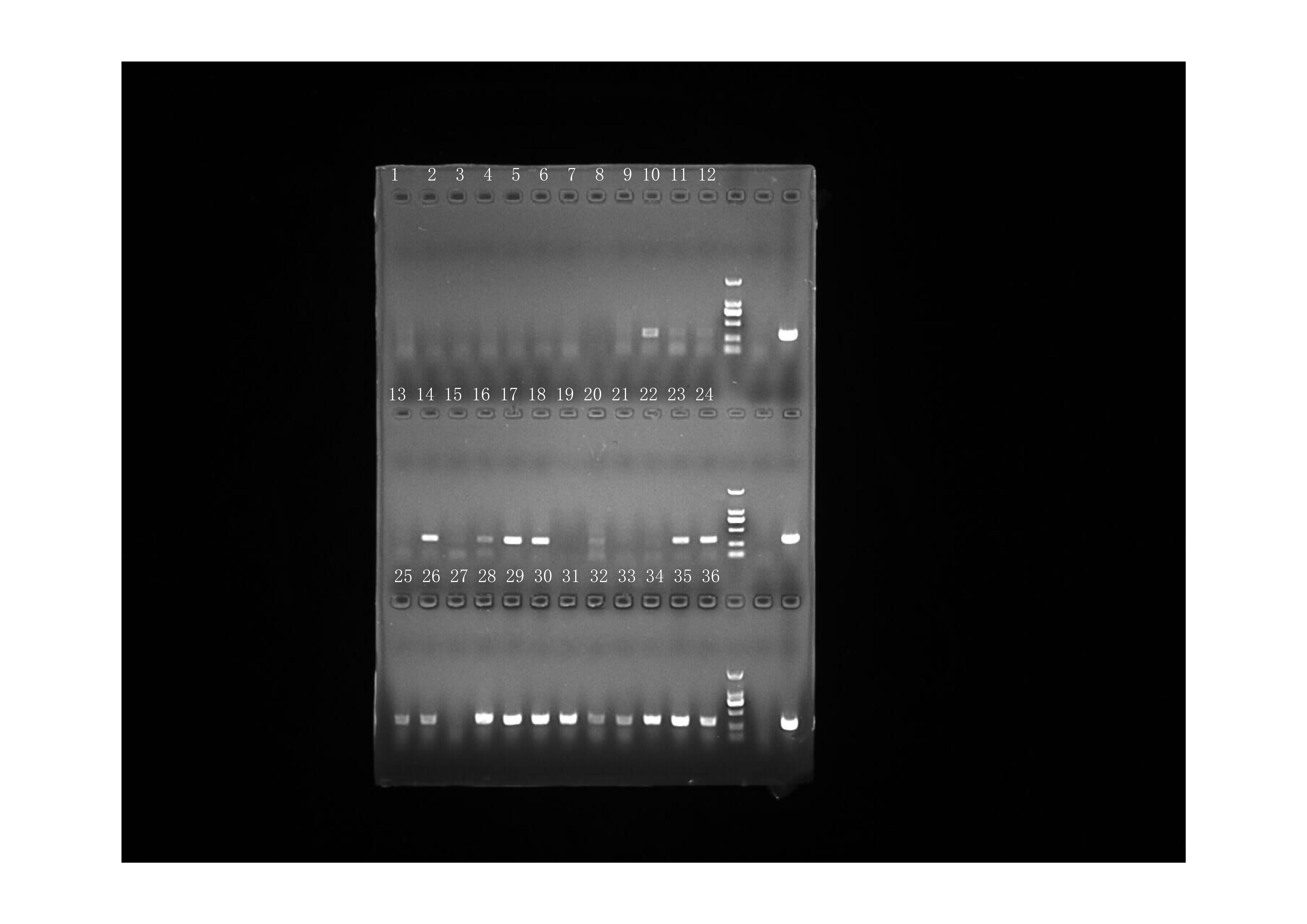

Supplement: Supplemental Information 10 — Raw data: result of RT-PCR amplificaiton of the NP gene resulting in a fragment of 330 bp. The name of sample was indicated in the picture. [file peerj-09-12512-s010.jpg]

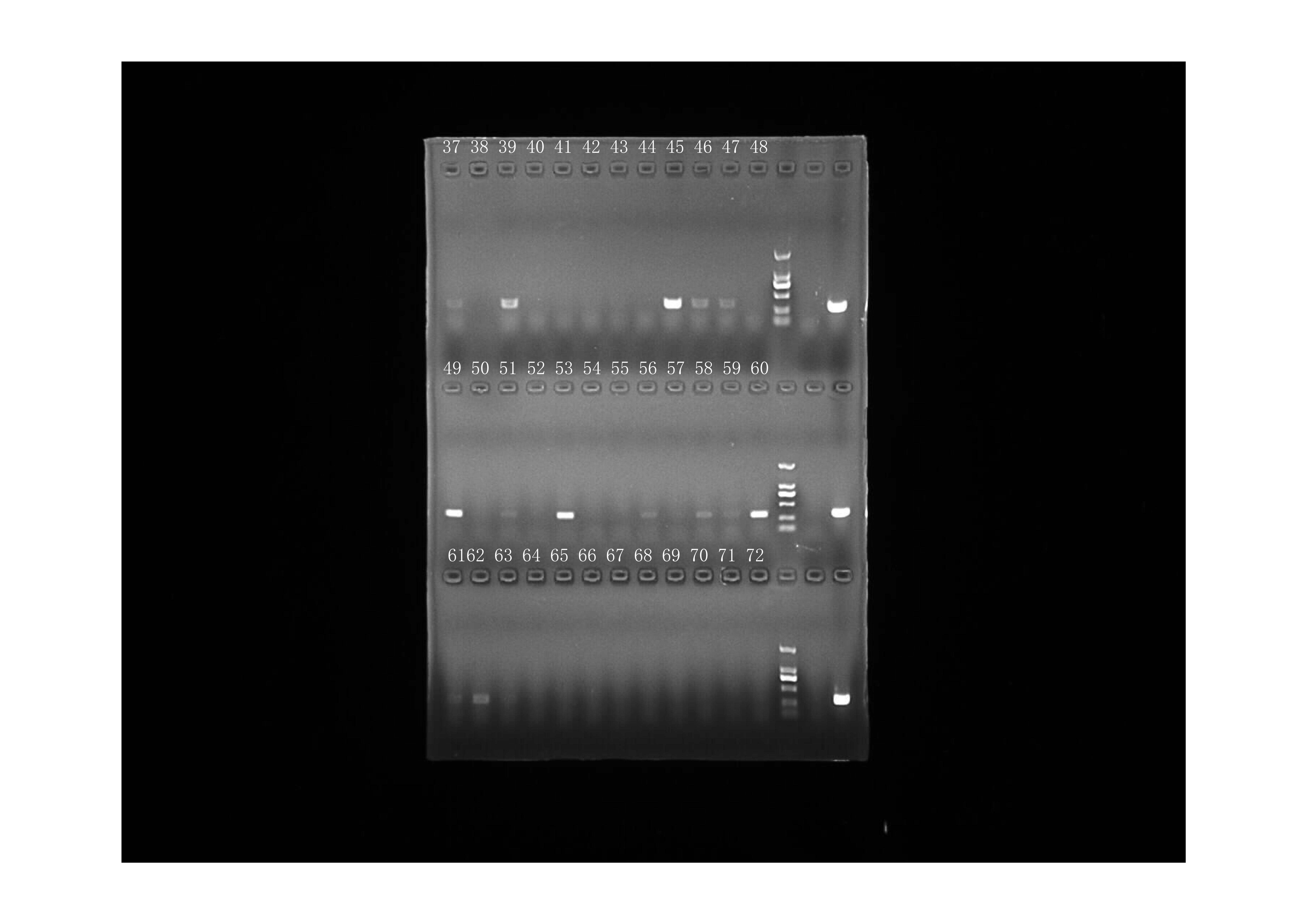

Supplement: Supplemental Information 11 — Raw data: result of RT-PCR amplificaiton of the NP gene resulting in a fragment of 330 bp. The name of sample was indicated in the picture. [file peerj-09-12512-s011.jpg]

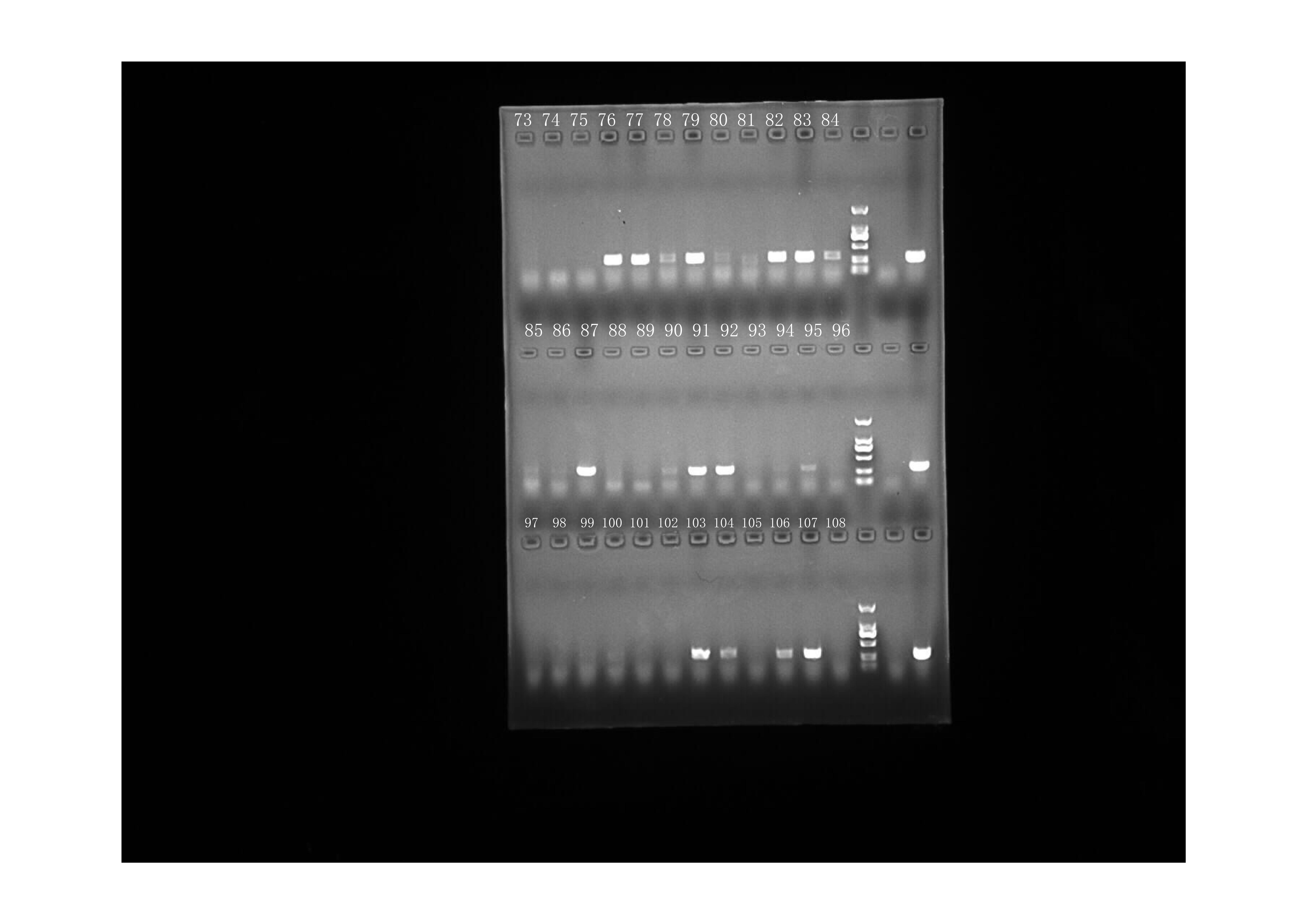

Supplement: Supplemental Information 12 — Raw data: result of RT-PCR amplificaiton of the NP gene resulting in a fragment of 330 bp. The name of sample was indicated in the picture. [file peerj-09-12512-s012.jpg]

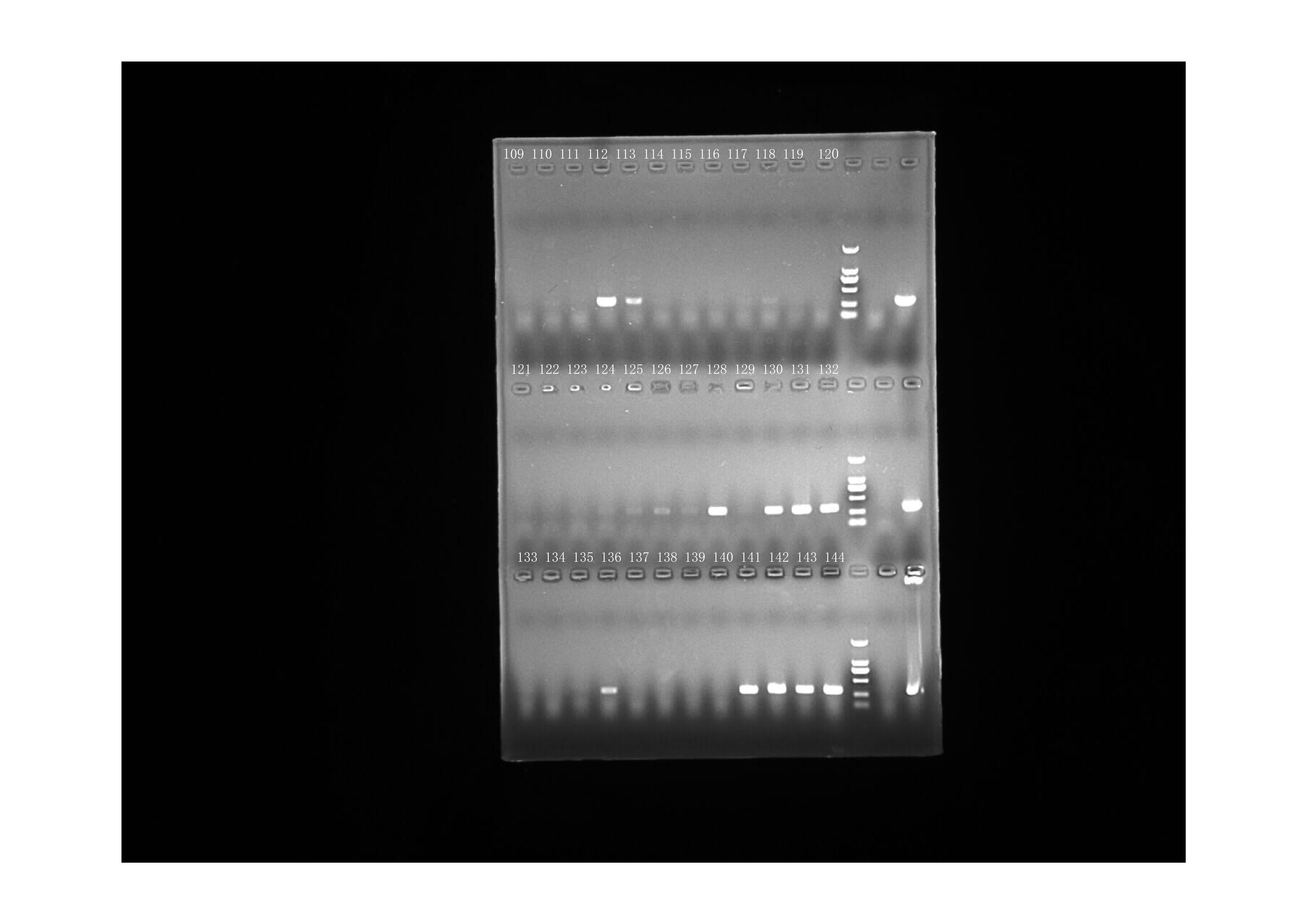

Supplement: Supplemental Information 13 — Raw data: result of RT-PCR amplificaiton of the NP gene resulting in a fragment of 330 bp. The name of sample was indicated in the picture. [file peerj-09-12512-s013.jpg]

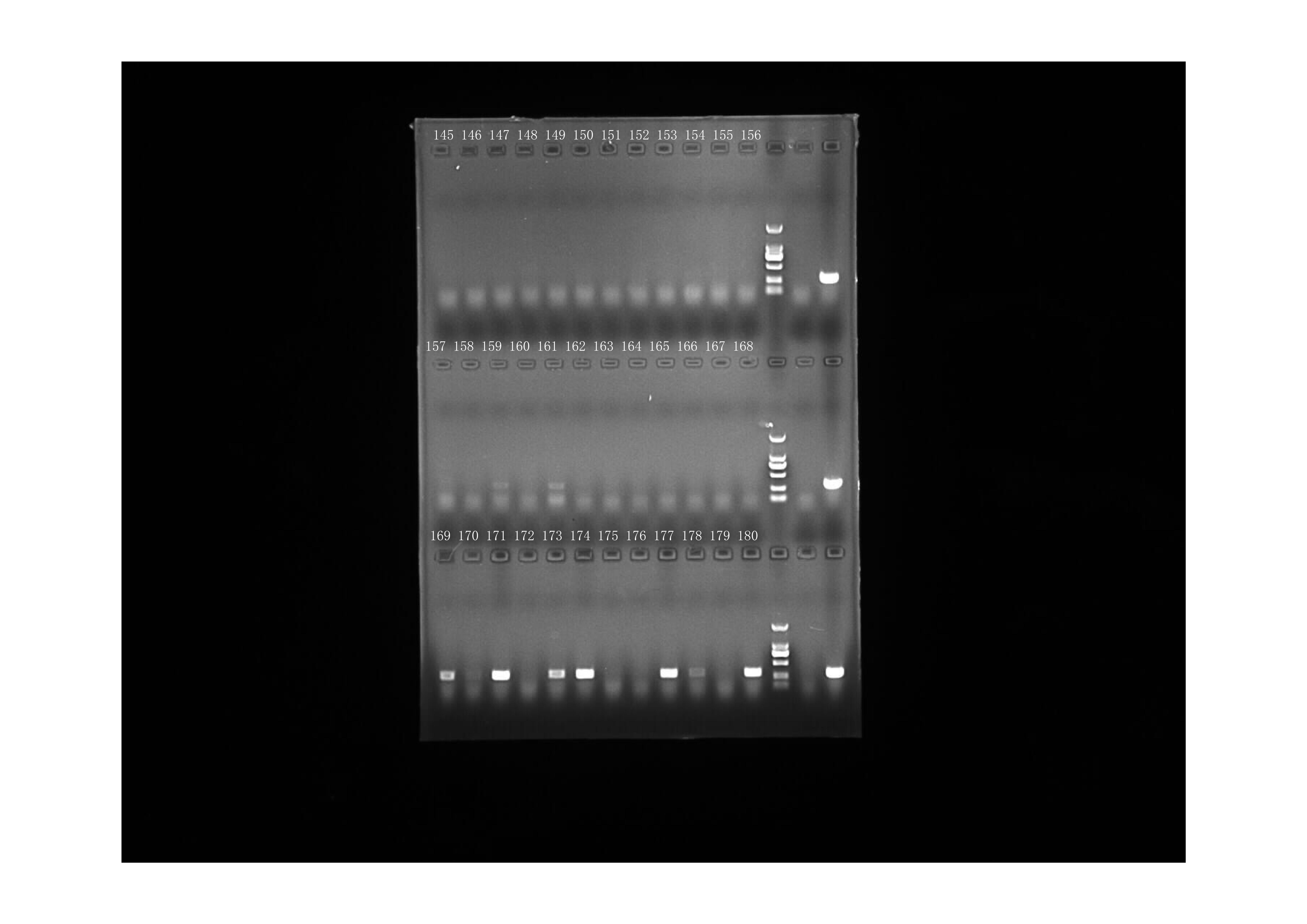

Supplement: Supplemental Information 14 — Raw data: result of RT-PCR amplificaiton of the NP gene resulting in a fragment of 330 bp. The name of sample was indicated in the picture. [file peerj-09-12512-s014.jpg]

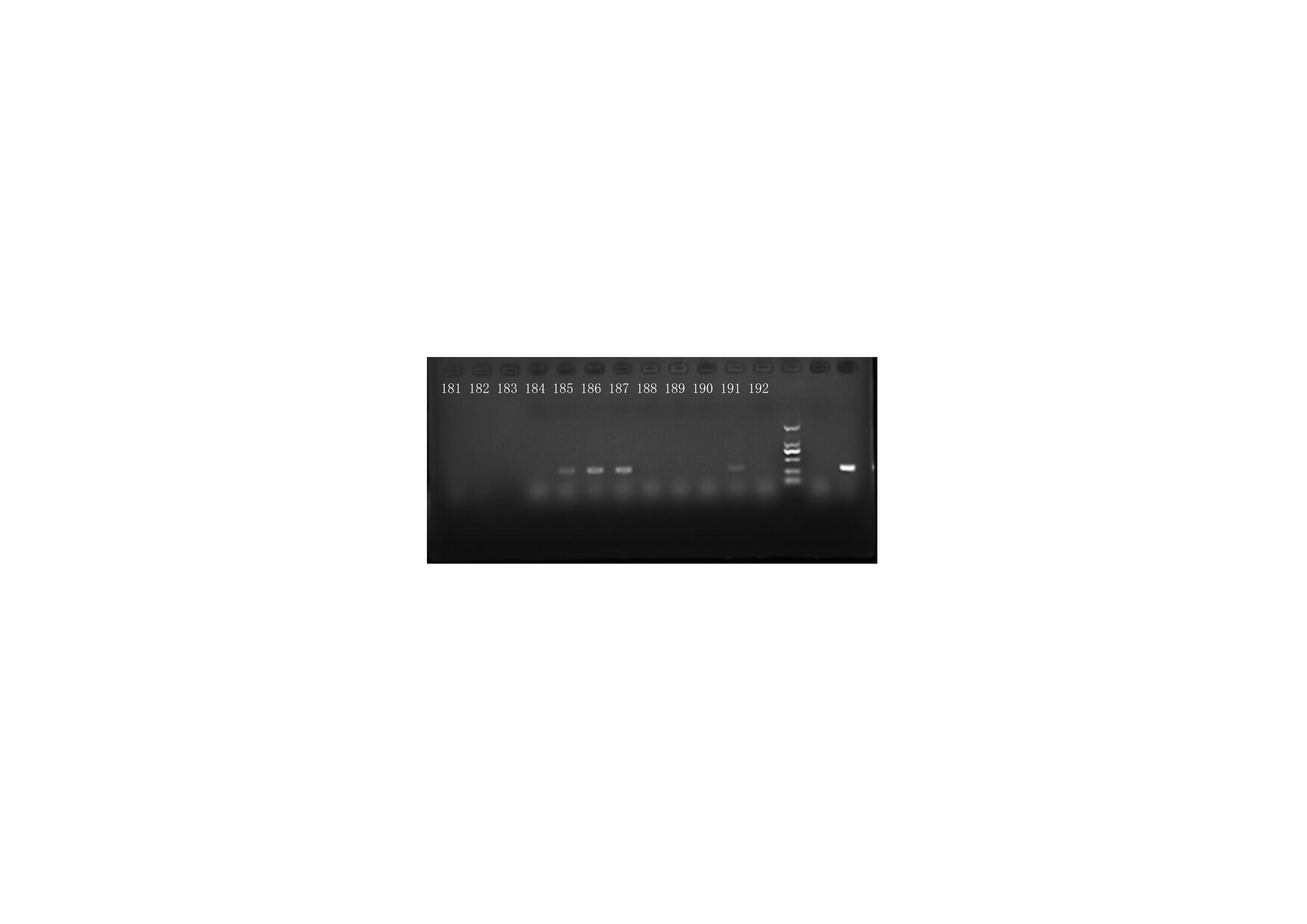

Supplement: Supplemental Information 15 — Raw data: result of RT-PCR amplificaiton of the NP gene resulting in a fragment of 330 bp. The name of sample was indicated in the picture. [file peerj-09-12512-s015.jpg]
