# Supplementary material for: Sequence characteristics and phylogenetic analysis of H9N2 subtype avian influenza A viruses detected from poultry and the environment in China, 2018
Source: PeerJ. 2021 Dec 20;9:e12512. doi: 10.7717/peerj.12512 (PMC8697764; doi:10.7717/peerj.12512)
Supplement: Supplemental Information 18 — The name and sequence of primers for amplification of HA and NA gene are indicated. In addition, the reaction system and thermoscycling are provided. [file peerj-09-12512-s018.pdf]

Primers used for RT-PCR

Name of primer	Sequence	Gene
Bm-HA-F	5′ -TATTCGTCTCAGGGAGCAAAGCAGGG-3′	HA
Bm-HA-R	5′ -ATATCGTCTCGTATTAGTAGAAACAAGGGTGTTTT-3′	
Ba-NA-F	5′ -TATTGGTCTCAGGGAGCAAAAGCAGGAGT-3′	NA
Ba-NA-R	5′ -ATATGGTCTCGTATTAGTAGAAACAAGGAGTTTTTT-3′	

Reaction system:

Premix *Taq*™	25 μL
AMV reverse transcriptase	0.4 μL
RNasin ribonuclease inhibitor	0.6 μL
Reverse primer and	0.1 μL
Forward primer	0.1 μL
Nuclease-free water	18.2 μL
RNA	4 μL

Reaction cycling:

42℃ for 45 min

95℃ for 3min

35 cycles:

- 94℃ for 30s
- 56℃ for 30s
- 68℃ for 2min 30s

72℃ for 10min
